# Multimessenger Binary Mergers Containing Neutron Stars: Gravitational Waves, Jets, and *γ*-Ray Bursts

**DOI:** 10.3389/fspas.2021.656907

**Published:** 2021-04-08

**Authors:** Milton Ruiz, Stuart L. Shapiro, Antonios Tsokaros

**Affiliations:** 1 Department of Physics, University of Illinois at Urbana-Champaign, Urbana, IL, United States,; 2 Department of Astronomy, University of Illinois at Urbana-Champaign, Urbana, IL, United States

**Keywords:** black holes, neutron stars, gravitational waves, short gamma-ray bursts, numerical relativity, multimessenger astronomy

## Abstract

Neutron stars (NSs) are extraordinary not only because they are the densest form of matter in the visible Universe but also because they can generate magnetic fields ten orders of magnitude larger than those currently constructed on earth. The combination of extreme gravity with the enormous electromagnetic (EM) fields gives rise to spectacular phenomena like those observed on August 2017 with the merger of a binary neutron star system, an event that generated a gravitational wave (GW) signal, a short *γ* -ray burst (sGRB), and a kilonova. This event serves as the highlight so far of the era of multimessenger astronomy. In this review, we present the current state of our theoretical understanding of compact binary mergers containing NSs as gleaned from the latest general relativistic magnetohydrodynamic simulations. Such mergers can lead to events like the one on August 2017, GW170817, and its EM counterparts, GRB 170817 and AT 2017gfo. In addition to exploring the GW emission from binary black hole-neutron star and neutron star-neutron star mergers, we also focus on their counterpart EM signals. In particular, we are interested in identifying the conditions under which a relativistic jet can be launched following these mergers. Such a jet is an essential feature of most sGRB models and provides the main conduit of energy from the central object to the outer radiation regions. Jet properties, including their lifetimes and Poynting luminosities, the effects of the initial magnetic field geometries and spins of the coalescing NSs, as well as their governing equation of state, are discussed. Lastly, we present our current understanding of how the Blandford-Znajek mechanism arises from merger remnants as the trigger for launching jets, if, when and how a horizon is necessary for this mechanism, and the possibility that it can turn on in magnetized neutron ergostars, which contain ergoregions, but no horizons.

## 1. INTRODUCTION

Gravitational wave astronomy was launched in 2015 with the first-ever gravitational wave (GW) detection of the inspiral and merger of a binary black hole (BHBH) system as reported by the LIGO/Virgo (LV) scientific collaboration—event GW150914 (Abbott et al., 2016a,b). Two years later the simultaneous detection of GWs from an inspiraling binary neutron star (NSNS) system, event GW170817, and its post-merger emission of electromagnetic (EM) radiation spurred the era of multimessenger astronomy (Abbott et al., 2017a,b,c,d; Kozlova et al., 2017). Although at present the LV scientific collaboration almost weekly announces new GW signals whose progenitors may be BHBHs, NSNSs, or black hole-neutron star (BHNS) systems there has been no robust discovery of a BHNS system yet, while the subsequent NSNS candidates have been EM “orphans” i.e., no EM radiation has been associated with the GWs produced by them. Merging NSNSs and BHNSs are not only important sources of gravitational radiation, but also promising candidates for coincident EM counterparts, which could give new insight into their sources. Namely, GWs are sensitive to the density profile of NSs and their measurement enforces tight constraints on the equation of state (EOS) that governs matter at supranuclear densities (Lattimer and Prakash, 2016), while postmerger EM signatures can help to explain the phenomenology of short *γ* -ray bursts (sGRBs), and nucleosynthesis processes powering kilonovae (Li and Paczynski, 1998; Metzger, 2017). To understand these observations and, in particular, to understand the physics of matter under extreme conditions, it is crucial to compare them to predictions from theoretical modeling, which, due to the complexity of the underlying physical phenomena, is largely numerical in nature.

Although a spinning BH surrounded by an accretion disk is the remnant of a BHNS merger, this is not necessarily the case for an NSNS merger. Depending on the total mass of the system, as well as the EOS of the NS companions, the outcome of an NSNS merger can be a stable NS or a spinning BH, surrounded by an accretion disk in either case. Even when a BH is the remnant, the path toward such an outcome is extremely varied and can be decisive for a number of important issues, like the existence of a sGRB or the production of the heaviest elements in the Universe via a kilonova (Metzger and Fernández, 2014). The current consensus for the event GW170817 is the formation of a transient NS remnant sustaining itself for a brief period of time ≲ 1 s before collapsing to a BH (this was inferred from the existence of a sGRB, and the large amount of ejecta ≳ 0.02 *M*_⊙_ estimated from the kilonova AT 2017gfo). Assuming that this was the case, it is possible to place strong constraints on the maximum mass of a cold spherical NS and its EOS (Margalit and Metzger, 2017; Shibata et al., 2017, 2019; Rezzolla et al., 2018; Ruiz et al., 2018a). These constraints could also provide an explanation for the unidentified 2.6 *M*_⊙_ compact object in GW190814 as a rotating or even a non-rotating NS (Most et al., 2020; Tsokaros et al., 2020a). From a different point of view, the absence of a prompt collapse scenario and the large ejecta mass also puts constraints on NS radii or, equivalently, their tidal deformability (Bauswein et al., 2017; Radice et al., 2018). These constraints on the NS radius coming directly from the postmerger object were further refined by complementary analyses of the GW inspiral signal, which can be used to estimate the tidal deformability of the inspiraling NSs (Abbott et al., 2017c; De et al., 2018; Raithel et al., 2018).

Lattimer and Schramm (1974) and Symbalisty and Schramm (1982) suggested that unstable neutron-rich nuclei can be built in the mergers of BHNS or NSNS systems through rapid neutron bombardment, the r-process. Apart from the dynamical ejecta that emerge within milliseconds after merger, the ejecta that emerge much later are very important in the determination of whether or not heavier elements through the r-process are being produced. Li and Paczynski (1998) argued that the low mass and high velocity of these ejecta will make them transparent to their own radiation, resulting in emission whose peak will last around 1 day. Metzger et al. (2010) calculated the luminosity of the radioactively-powered transients in NS mergers and found these transients to be approximately 1,000 times brighter than typical novae, therefore calling them “kilonovae.” Metzger and Fernández (2014) argued that the lifetime of the merger remnant is directly imprinted in their early “blue” emission (from high electron fraction, lanthanide-poor ejecta) or late “red” emission (from low electron fraction, lanthanide-rich ejecta), both of which have been seen in event GW170817. The blue emission suggested ejecta composed of light r-process elements, while the red emission is consistent with heavier ones (lanthanide or actinides). The overall conclusion is the kilonova AT 2017gfo was a major source of r-process elements (Côté et al., 2017; Kasen et al., 2017).

Another important characteristic associated with event GW170817 was the observation of an sGRB—event GRB 170817A (Abbott et al., 2017b; Kozlova et al., 2017). This GRB was unusually weak, and various models have been proposed to explain this, including a choked-jet cocoon or a successful-jet cocoon (Hallinan et al., 2017; Kasliwal et al., 2017; Mooley et al., 2018). Recently, Mooley et al. (2018) using radio observations from very long-baseline interferometry were able to break the degeneracy between the choked and successful-jet cocoon models and concluded that the early-time radio emission was powered by a wide-angle outflow (a cocoon), while the late-time emission was most probably dominated by an energetic and narrowly collimated jet with an opening angle of <5°, and observed from a viewing angle of about 20°. This solidified theoretical predictions that NSNS, or at least a stellar binary where at least one of the companions is a NS, can be the progenitors of the central engine that power sGRBs (Paczynski, 1986; Eichler et al., 1989; Narayan et al., 1992).

Although GRB 170817A provided the long-sought observational evidence linking sGRBs with NSNS mergers, it did not reveal the nature of the central engine behind the launching of a relativistic jet. In particular, is a BH horizon necessary for the existence of a jet or is it just sufficient (Paschalidis et al., 2015; Ruiz et al., 2016, 2018b, 2019; Ruiz and Shapiro, 2017)? If necessary, then a stable NS remnant cannot be the generator of such jets. If not, is the jet from a stable NS qualitatively the same as the one launched from a spinning BH immersed in a gaseous disk? In particular, can one describe it as a Blandford and Znajek (1977) (BZ) jet? Notice that according to Komissarov (2002, 2004, 2005) and Ruiz et al. (2012), the driving mechanism behind a BZ jet is not the horizon but the ergoregion. Thus, while it may be that typical NSs cannot launch a BZ jet, NSs that contain ergoregions—ergostars—might be able to Ruiz et al. (2020c).

Since the pioneering general relativity (GR) simulations of NSNS mergers by Shibata and Uryu (2000) and BHNS mergers by Baumgarte et al. (2004), Shibata and Uryu (2006), and Faber et al. (2006a,b), a number of groups have produced a large body of work that captures the main characteristics of such events (see reviews by Shibata and Taniguchi, 2011; Baiotti and Rezzolla, 2017; Foucart, 2020). Below we will present a brief review of some of the important progress in the field, paying special attention to pure hydrodynamical vs. magnetohydrodynamical simulations. Details regarding the techniques used (either in evolution or in the initial data) will be omitted. We refer the reader to e.g., Alcubierre (2008), Baumgarte and Shapiro (2010), Shibata (2015) for such details. We also do not treat white dwarf-neutron star (WDNS) mergers, which, though important for GW detections by LISA, are not likely sources of sGRBs or kilonova. We refer readers interested to the GR simulations of Paschalidis et al. (2011) and references therein.

We adopt geometrized units with *c* = *G* = 1 unless otherwise indicated.

## 2. BLACK HOLE-NEUTRON STARMERGERS: REMNANTS AND INCIPIENT JETS

Motivated by the significance of BHNS binaries as copious sources of GW and EM radiation, many numerical studies have been performed over the past years. Before the pioneering BHBH simulations (Pretorius, 2005; Baker et al., 2006; Campanelli et al., 2006), most dynamical simulations of BHNS binaries were treated in Newtonian gravity, modeling the BH as a point mass (Lee, 2001; Kobayashi et al., 2004; Rosswog et al., 2004; Rosswog, 2005; Rantsiou et al., 2008). Although these studies gave first insights on the basic dynamics of BHNSs, full GR simulations are required to properly model the late inspiral, NS disruption, tidal tails, merger remnant, disk mass, fraction of unbound material ejected, sGRB engine, and most significantly the GWs emitted during merger. In the following section, we only review full GR studies of these binaries.

### 2.1. Nonmagnetized Evolutions

Most of the close BHNS binary orbits are likely quasi-circular, since gravitational radiation reduces the orbital eccentricity of the binary as it evolves toward smaller orbits (Peters, 1964). However, a small fraction may form in dense stellar regions, such as globular cluster or galactic nuclei, through dynamical capture, and they may merge with high eccentricities (Lee et al., 2010; Kocsis and Levin, 2012; Samsing et al., 2014).

Motivated by the above, different groups have generated quasi-equilibrium initial data for BHNSs on quasi-circular orbits (Baumgarte et al., 2004; Taniguchi et al., 2005, 2007; Grandclement, 2006; Shibata and Uryu, 2006, 2007; Foucart et al., 2008). Some of the earliest full GR simulations of these configurations were performed by Shibata and Uryu (2006, 2007), followed by Etienne et al. (2008) and Duez et al. (2008). In all of these studies the binary was formed by a nonspinning BH with a NS companion modeled as a Γ = 2 polytrope. These simulations showed that the fate of BHNS remnants can be classified in two basic categories: (1) the NS is tidally disrupted before reaching the innermost stable circular orbit (ISCO), inducing a long tidal tail of matter that eventually wraps around the BH and forms a significant accretion disk (typically with a mass ≳ 8% of the NS rest-mass); (2) the NS plunges into the BH, leaving a BH surrounded by a negligibly small accretion disk (typically with a mass ≲ 2% of the NS rest-mass).

Using a Smoothed Particle Hydrodynamics (SPH) code and an approximate “conformal” GR metric, Rantsiou et al. (2008) showed that the mass of the accretion disk remnant strongly depends on the magnitude and direction of the BH spin. In particular, it was found that only systems with a highly spinning BH, and slightly misaligned to the total angular of the system, yield significant accretion disk remnants. These results were later confirmed by full GR studies (Etienne et al., 2009; Foucart et al., 2011, 2012; Kyutoku et al., 2011) showing that for sufficiently high BH spins, mass ratios *q* = *M*_BH_/*M*_NS_ ≲ 3, and/or lower NS compactions $C=M_{\text{NS}}/{\text{R}}_{\text{NS}}≲0.18$, a substantial disk can form following merger. Here *M*_BH_ is the Christodoulou (1970) BH mass at infinite separation and *M*_NS_ the NS rest mass, while $M_{\text{NS}}$ and *R*_NS_ are the gravitational [Arnowitt-Deser-Misner (ADM)] mass and the circumferential radius of the star in isolation, respectively.

Using the above numerical simulation results, Foucart (2012) constructed a simple fitting formula to predict the amount of matter remaining outside the BH horizon about 10 ms following merger:

(1) $$\frac{M_{\text{disk}}}{M_{\text{NS}}}\approx 0.415q^{1/3}(1-2C)-0.148\frac{R_{\text{ISCO}}}{R_{\text{NS}}}.$$

This expression is valid for mass ratios in the range *q* = 3–7, BH spins *a*_BH_/*M*_BH_ = 0–0.9, and NSs with radii *R*_NS_ = 11–16 km, thereby encompassing the most likely astrophysically relevant parameter space. Here, *M*_disk_ and *R*_ISCO_ is the mass of the disk remnant and the radius of the ISCO, respectively. Note that Equation (1) explicitly shows that the mass of the disk remnant depends on the EOS and the BH spin, which determine the mass and radius of the NS and the position of the ISCO, respectively. It should be noticed that BHNSs with nearly-extremal BH spins have been considered by Lovelace et al. (2008), Lovelace et al. (2013). These studies found that upon NS disruption, less than half of the matter is promptly accreted by the BH, around 20% becomes unbound and escapes, and the remaining mass settles into a massive accretion disk.

Early population synthesis studies found that the distribution of mass ratios in BHNSs depends on the metallicity and peaks at *q* = 7 (Belczynski et al., 2008, 2010), but more recent works found that it is generally <10, peaking at *q* ≈ 5 (Giacobbo and Mapelli, 2018; Abbott et al., 2020a). Using Equation (1), one finds that, for a binary with mass ratio *q* = 5 in which the NS companion has radius 13.3 km and rest-mass *M*_NS_ = 1.44 *M*_⊙_ (compatible with NICER observations; Miller et al., 2019; Riley et al., 2019) a BH spin of *a*_BH_/*M*_BH_ ≳ 0.65 is required to form an accretion disk with ≳ 10% of the NS rest mass. The power available for EM emission is usually taken to be proportional to the accretion rate. Under this assumption, it is expected that the luminosity of the disk remnant is $L_{\text{EM}}=\epsilon {\dot{M}}_{\text{disk}}$, where *ϵ* is the efficiency for converting accretion power to EM luminosity and ${\dot{M}}_{\text{disk }}\sim M_{\text{disk}}/t_{\text{acc}}$ is the rest-mass accretion rate, where *t*_acc_ is the disk lifetime. Assuming a 1% efficiency and a disk lifetime of ~ 0.2 s, the luminosity is *L*_EM_ ~ 10^51^ erg/s, consistent with typical EM luminosities of sGRBs. This value is also consistent with the “universal” merger scenario for generating EM emission from merger and collapse BH + disk remnants (Shapiro, 2017). These results allow us to conclude that the merger of NSs orbiting highly spinning BHs can be the progenitors of the engines that power sGRBs. However, the LV scientific collaboration has reported the observation of BHBHs having high mass and/or low spins (see e.g., Table VI in Abbott et al., 2020b). If this trend continues for LV-like BHNSs, then it is expected that LV-like BHNS remnants would have negligible accretion disks, which might disfavor their role as progenitors of sGRBs and/or observable kilonovae.

The previous numerical studies assumed that the NS companion is irrotational. Recently, East et al. (2015) and Ruiz et al. (2020a) showed that the NS spin has a strong impact on the disk remnant and the dynamical ejecta. As the prograde NS spin increases, the effective ISCO decreases (Barausse and Buonanno, 2010). In addition, as the magnitude of the NS spin increases, the star becomes less bound and the tidal separation radius *r*_tid_ (separation at which tidal disruption begins) increases, also resulting in more pronounced disruption effects. This effect can be easily understood by estimating *r*_tid_ by equating the inward gravitational force exerted by the NS on its fluid elements with the BH’s outward tidal and the outgoing centrifugal forces to obtain

(2) $$r_{\text{tid}}/M_{\text{BH}}≃q^{-2/3}C^{-1}{\left[1-\Omega^{2}M_{NS}^{2}C^{-3}\right]}^{-1/3},$$

(Ruiz et al., 2020a) where Ω = *a*_NS_*M*_NS_/*I*. Here *a*_NS_ is the NS spin parameter and *I* its moment of inertia. This simple Newtonian expression shows that the larger the mass ratio and/or the compaction of the NS, the closer the tidal separation to the ISCO. The NS then experiences tidal disruption effects only during a short time before the bulk of the NS plunges onto the BH. In contrast, the larger the magnitude of the NS spin, the farther away *r*_tid_ is from the ISCO. In this case, the star can be tidally disrupted before being swallowed by the BH which increases the time for disruption and with it the amount of matter that spreads out to form the disk or escapes to infinity.

Recently, Barnes and Kasen (2013) showed that the opacities in r-process ejecta are likely dominated by lanthanides, which induce peak bolometric luminosities for kilonovae of

(3) $$L_{\text{knova}}\approx 10^{41}{\left(\frac{M_{\text{e}j\text{e}}}{10^{-2}M_{⊙}}\right)}^{1/2}{\left(\frac{{\text{v}}_{\text{e}j\text{e}}}{0.3\text{c}}\right)}^{1/2}\text{erg}/\text{s,}$$

(East et al., 2015) and rise times of

(4) $$t_{\text{peak }}\approx 0.25{\left(\frac{M_{\text{eje}}}{10^{-2}M_{⊙}}\right)}^{1/2}{\left(\frac{{\text{v}}_{\text{eje}}}{0.3\text{c}}\right)}^{-1/2}\text{ days },$$

(East et al., 2015) where v_eje_ and *M*_eje_ are the mass-averaged velocity and rest-mass of the ejecta. The characteristic speed of the ejecta is v_eje_/*c* ≲ 0.2–0.3 with a rest-mass of ≲ 10^−3^ *M*_⊙_ (see e.g., East et al., 2015; Foucart et al., 2015; Hayashi et al., 2020; Ruiz et al., 2020a). Therefore, the bolometric luminosity of kilonova signals is *L*_knova_ ≲ 10^41^ erg/s with rise times of ≲ 7 h. These luminosities correspond to an R band magnitude of ~ 24 mag at 200 Mpc inside the aLIGO volume (Abbott et al., 2013), and above the LSST survey sensitivity of 24.5 mag (Barnes and Kasen, 2013; East et al., 2015). Hence some of these signals may be detectable by the LSST survey (Ruiz et al., 2020a).

It is worth noting that BHs and white dwarfs can also form binaries that undergo merger and eventually form a BH + disk remnant (see e.g., Nelemans et al., 2001; McKernan et al., 2020). These binaries are expected to be formed in dense star regions such as in galactic centers and globular clusters. Newtonian SPH calculations by Fryer et al. (1999) suggested that BH + massive WD binary mergers can release energies of 10^48^ − 10^51^ erg that may explain typical long duration GRBs, though population synthesis studies predict a (low) merger rate of 1.91^6^ yr − 1 (see e.g., Nelemans et al., 2001).

### 2.2. Magnetized Evolutions

The previous numerical studies showed that BHNS mergers can create the right conditions to power sGRBs (i.e., a spinning BH + disk). However, they do not account for either magnetic fields or neutrino pair annihilation processes, the most popular components invoked in most sGRB models to drive jets (see e.g., Blandford and Znajek, 1977; Vlahakis and Konigl, 2003; Aloy et al., 2004; Piran, 2005). As the lifetime of the neutrino pair annihilation process might be too small to explain typical sGRBs (Kyutoku et al., 2018), we henceforth focus only on the magnetic process. However, it is worth noting that BH + disk remnants powering sGRBs may be dominated initially by neutrino pair annihilation processes followed by the BZ mechanism (Dirirsa, 2017), leading to a transition from a thermally-dominated fireball to a Poynting EM-dominated flow, as is inferred for some GRBs, such as GRB 160625B (Zhang et al., 2018).

Ideal GR magnetohydrodynamics (GRMHD) studies of magnetized BHNS mergers in which the NS is initially endowed with an *interior-only* poloidal magnetic field generated by the vector potential

(5) $$\begin{array}{l} A_{i}=\left(-\frac{y-y_{c}}{\varpi_{c}^{2}}\delta^{x}i+\frac{x-x_{\text{c}}}{\varpi_{c}^{2}}\delta^{y}{}_{i}\right)A_{\varphi },\\ A_{\varphi }=A_{b}\varpi_{c}^{2}\text{ max}{\left(P-P_{\text{cut}},0\right)}^{n_{b}}, \end{array}$$

were carried out by Chawla et al. (2010), Etienne et al. (2012a), and Kiuchi et al. (2015b), varying the mass ratio, the BH spin, and the strength of the magnetic field. Here the orbital plane is at *z* = 0, (*x*_c_, *y*_c_, 0) is the coordinate location of the center of mass of the NS, $\varpi_{c}^{2}={\left(x-x_{c}\right)}^{2}+{\left(y-y_{c}\right)}^{2}$, and *A*_*b*_, *n*_*p*_, and *P*_cut_ are free parameters. The cutoff pressure parameter *P*_cut_ confines the magnetic field inside the NS within *P* > *P*_cut_. The parameter *n*_*b*_ determines the degree of central condensation of the magnetic field.

**FIGURE 1 |:**
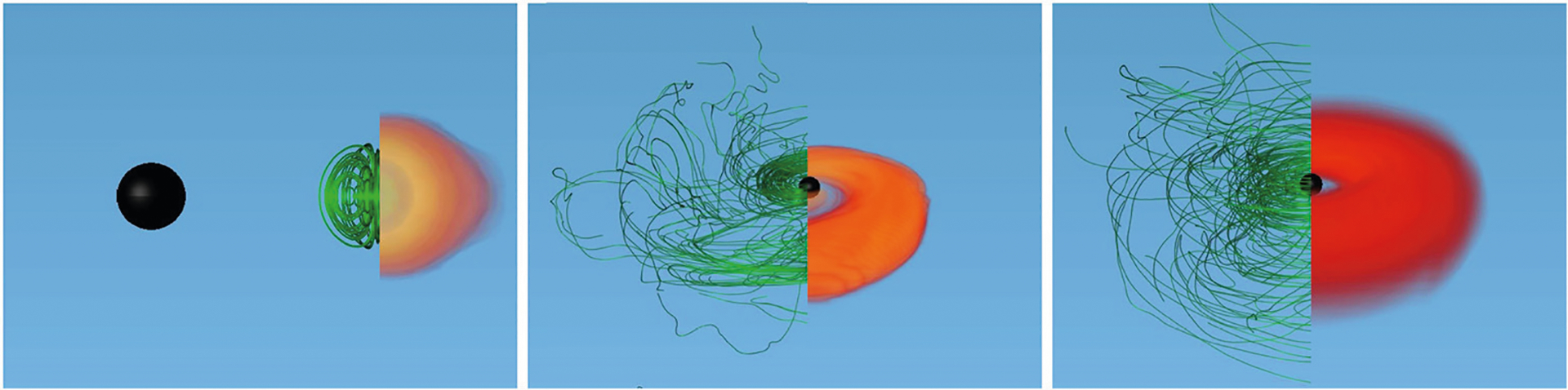
The NS magnetic field lines (green) and rest-mass density *ρ*_0_ (reddish) normalized to the initial NS maximum value *ρ*_0_ = 8.92 × 10^14^ (1.4*M*_⊙_/*M*_NS_)^2^g /cm^3^, at selected times for a BHNS with mass ratio *q* = 3. The initial BH spin is *a*_BH_/*M*_BH_ = 0.75 and the NS is an irrotational Γ = 2 polytrope. Here the BH apparent horizon is shown as a black sphere. Following merger, the field lines are wound into an almost purely toroidal configuration (adapted from Etienne et al., 2012b).

**FIGURE 2 |:**
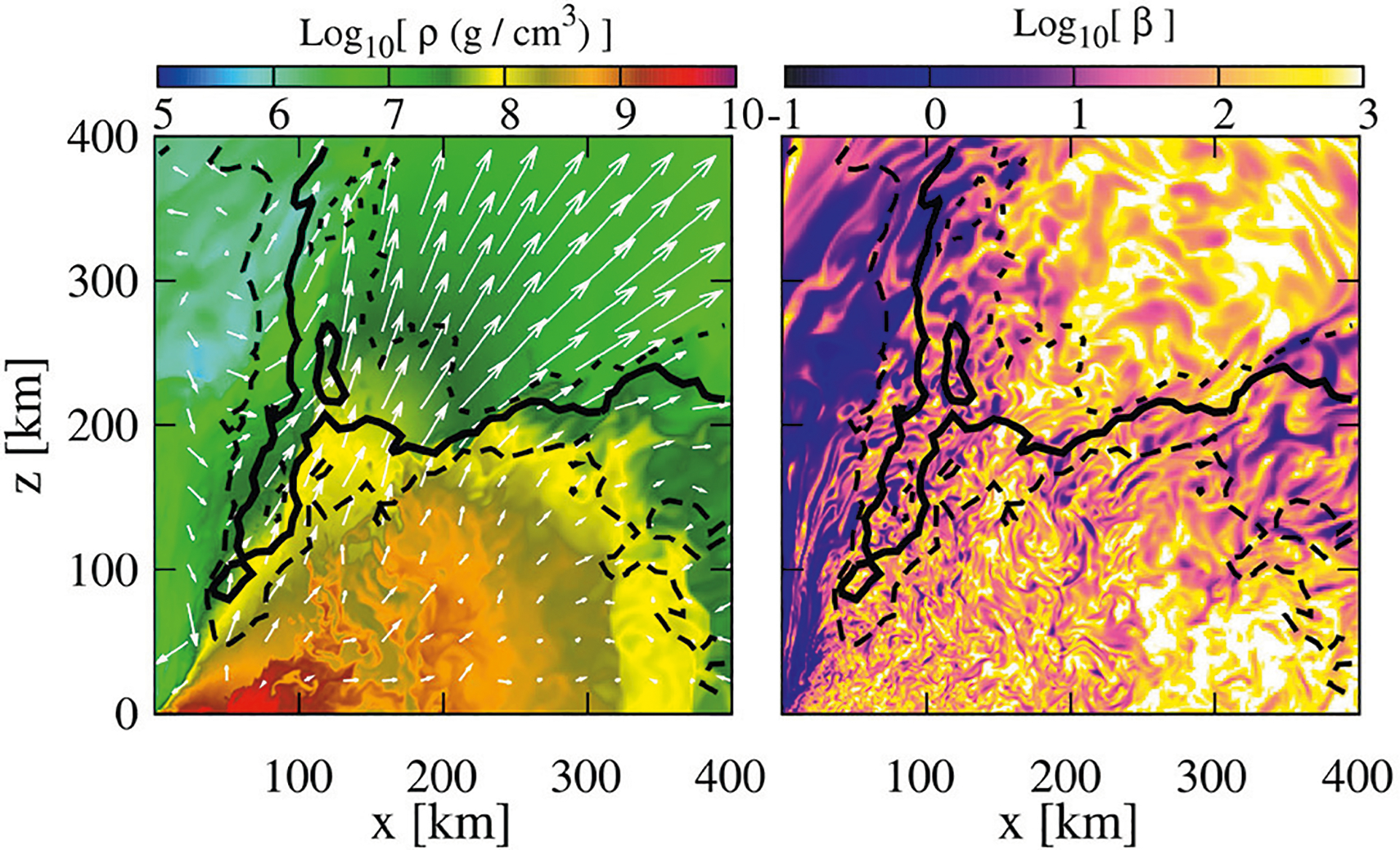
NS rest-mass density with fluid velocity arrows **(left)** and the gas-to-magnetic-pressure ratio **(right)** of a *q* = 4 BHNS remnant after ~ 50 ms following merger. A thermally-driven wind (but no collimated) outflow is observed (from Kiuchi et al., 2015b).

These numerical simulations showed that following merger, tidal tails of matter wrap around the BH, forming the accretion disk and dragging the frozen-in magnetic field into an almost purely toroidal configuration (see Figure 1). These simulations did not find any evidence of jet launching following the BH + disk formation. Nevertheless, Kiuchi et al. (2015b) reported that in their high-resolution simulations, in which the mass ratio is *q* = 4, the BH has a spin *a*_BH_/*M*_BH_ = 0.75, and the NS is modeled by the APR EOS (Akmal et al., 1998), a thermally-driven wind (but no collimated) outflow emerges after ~ 50 ms following merger (see Figure 2).

The lack of magnetically-driven jets in these simulations has been attributed to the fact that the magnetic field in the disk remnant is almost purely *toroidal*. Beckwith et al. (2008) showed that BH + disk systems can launch and support magnetically-driven jets only if a net *poloidal* magnetic flux is accreted onto the BH. Motivated by this conclusion, Etienne et al. (2012b) endowed the disk remnant from an unmagnetized BHNS simulation with a purely poloidal field and found that, indeed, under the *right conditions*, a jet can be launched from BHNS remnants. However, identifying the initial configuration of the seed magnetic field in the NS prior to tidal disruption that could lead to these conditions remained elusive for many years.

Paschalidis et al. (2015) then demonstrated that a more realistic initial magnetic configuration for the NS companion—a dipolar magnetic field extending from the NS interior into the *exterior* (as in pulsars)—could do the trick. Such a field can be generated by the vector potential

(6) $$A_{\phi }=\frac{\pi \;\varpi^{2}\;I_{0}\;r_{0}^{2}}{{\left(r_{0}^{2}+r^{2}\right)}^{3/2}}\left[1+\frac{15r_{0}^{2}\left(r_{0}^{2}+\varpi^{2}\right)}{8{\left(r_{0}^{2}+r^{2}\right)}^{2}}\right],$$

(Paschalidis et al., 2013) which approximately corresponds to a vector potential generated by an interior current loop. Here *r*_0_ is the current loop radius, *I*_0_ is the current, and $r^{2}=\varpi^{2}+z^{2}$, with $\varpi^{2}={\left(x-x_{\text{c}}\right)}^{2}+{\left(y-y_{\text{c}}\right)}^{2}$. To reliably evolve the exterior magnetic field with an ideal GRMHD code and simultaneously mimic the magnetic-pressure dominant environment that characterizes a pulsar-like magnetosphere, a low and variable density atmosphere was installed initially in the exterior where magnetic field stresses dominate over the fluid pressure.

**FIGURE 3 |:**
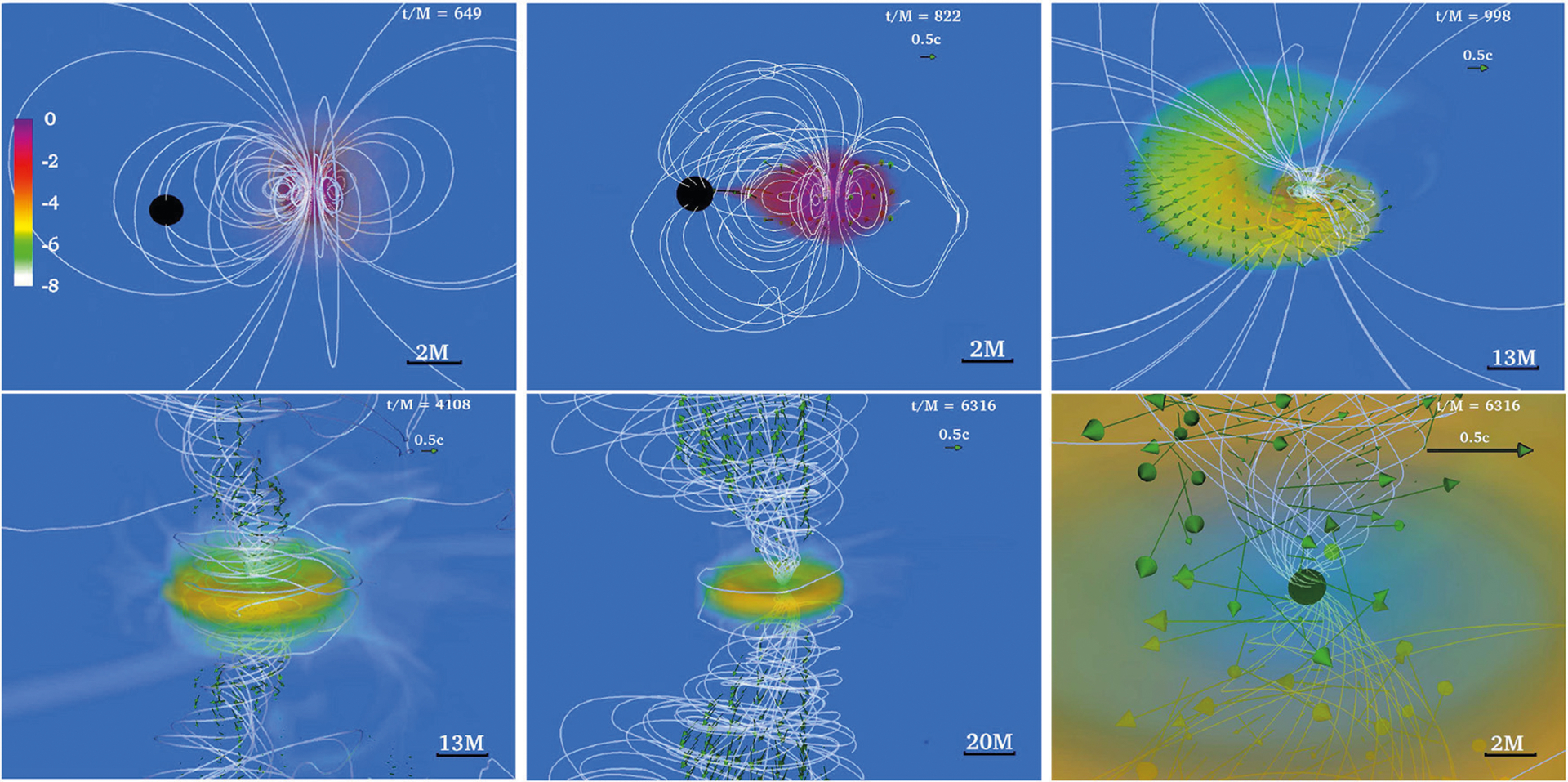
NS rest-mass density *ρ*_0_ normalized to its initial maximum value *ρ*_0,max_ = 8.92 × 10^14^(1.4*M*_⊙_/*M*_NS_)^2^ g/cm^3^ (log scale) at selected times for a BHNS with mass ratio *q* = 3. The initial BH spin is *a*_BH_/*M*_BH_ = 0.75 and the NS is an irrotational Γ = 2 polytrope. Arrows indicate fluid velocities and white lines the magnetic field lines. Bottom panel shows the system after an incipient jet is launched. Here M = 2.5 × 10^−2^ (*M*_NS_/1.4*M*_⊙_) ms = 7.58(*M*_NS_/1.4M_⊙_) km (from Paschalidis et al., 2015).

The above technique was used by Paschalidis et al. (2015) and Ruiz et al. (2018b, 2020a) to perform a series of BHNS simulations varying the density of the “artificial” atmosphere, the binary mass-ratio, the BH and NS spins, and the orientation of the seed magnetic field axis with respect to the orbital angular momentum. It was found that independent of the atmosphere or the NS spin, a magnetically driven, incipient jet is launched once the regions above the BH poles become nearly force-free (*B*^2^/8 *πρ*_0_ ≫ 1) for small tilt-angle magnetic fields and binary mass ratios that yield a significant disk remnant. The jet is confined by a collimated, tightly wound, helical magnetic funnel above the BH poles. Following the onset of accretion, the magnetic field in the disk remains predominantly toroidal as in the previous simulations. However, the external magnetic field maintains a strong poloidal component that retains footpoints at the BH poles. Magnetic instabilities [mainly magnetic winding and magnetorotational (MRI)] amplify the magnetic field from ~ 10^13^(1.4*M*_⊙_/*M*NS) G to ~ 10^15^(1.4*M*_⊙_/*M*NS) G at the BH poles, and after Δ*t* ~ 90 − 150(*M*_NS_/1.4*M*_⊙_) ms following merger a bonafide jet finally emerges (see Figure 3). It is worth noting that the calculation of Ruiz et al. (2020a) showed that the larger the initial NS prograde spin, the larger the mass of the accretion disk remnant. Similar behavior was observed for the amount of unbound ejecta. These results suggest that moderately high-mass ratio BHNSs (*q* ≲ 5) that undergo merger, where the NS companion has a non-negligible spin, may give rise to detectable kilonovae even if magnetically-driven jets are not formed.

The Lorentz factor in the funnel is Γ_L_ ~ 1.2 − 1.3, and hence the jet just above the BH poles is only mildly relativistic. However, the maximum attainable Lorentz factor of a magnetically—powered, nearly axisymmetric jet is comparable to the force-free parameter *B*^2^/8*πρ*_0_ inside the funnel (Vlahakis and Konigl, 2003). Near the end of the simulations the force-free parameter in the funnel reaches values ≳ 100. Thus, it is expected that the jet will be accelerated to Γ_L_ ≳ 100 as required by most sGRB models (Zou and Piran, 2010).

The lifetime of the disk is Δ*t* ~ 500(*M*_NS_/1.4*M*_⊙_) −700(*M*_NS_/1.4*M*_⊙_) ms and the outgoing EM Poynting luminosity is *L*_EM_ ~ 10^51±1^erg/s, and hence consistent with typical sGRBs (Bhat et al., 2016; Lien et al., 2016; Svinkin et al., 2016; Ajello et al., 2019). The luminosity is also consistent with that generated by the BZ mechanism

(7) $$L_{\text{BZ}}\sim 10^{51}{\left(\frac{a_{\text{BH}}}{M_{\text{BH}}}\right)}^{2}{\left(\frac{M_{\text{BH}}}{5.6M_{⊙}}\right)}^{2}{\left(\frac{B}{10^{15}\text{G}}\right)}^{2}\text{erg}/\text{s},$$

(Thorne et al., 1986) as well as with the simple analytic model that seems to apply universally for typically compact binaries mergers containing magnetized NSs that leave BH + disk remnants (Shapiro, 2017).

The above results were obtained with a high initial magnetic field. Paschalidis et al. (2015) argued that a smaller initial field will yield the same qualitative outcome because the magnetic field amplification following disruption is due largely to magnetic winding and the MRI. Amplification proceeds until appreciable differential rotational and internal energy of the plasma in the disk has been converted to magnetic energy. This amplification yields *B* ~ 10^15^*G* at the BH poles nearly independent of the initial NS magnetic field. Winding occurs on an Alfvén timescale, so amplification may take longer the weaker the initial field.

### 2.3. GW190814: Spin and EOS for a NS Companion

**FIGURE 4 |:**
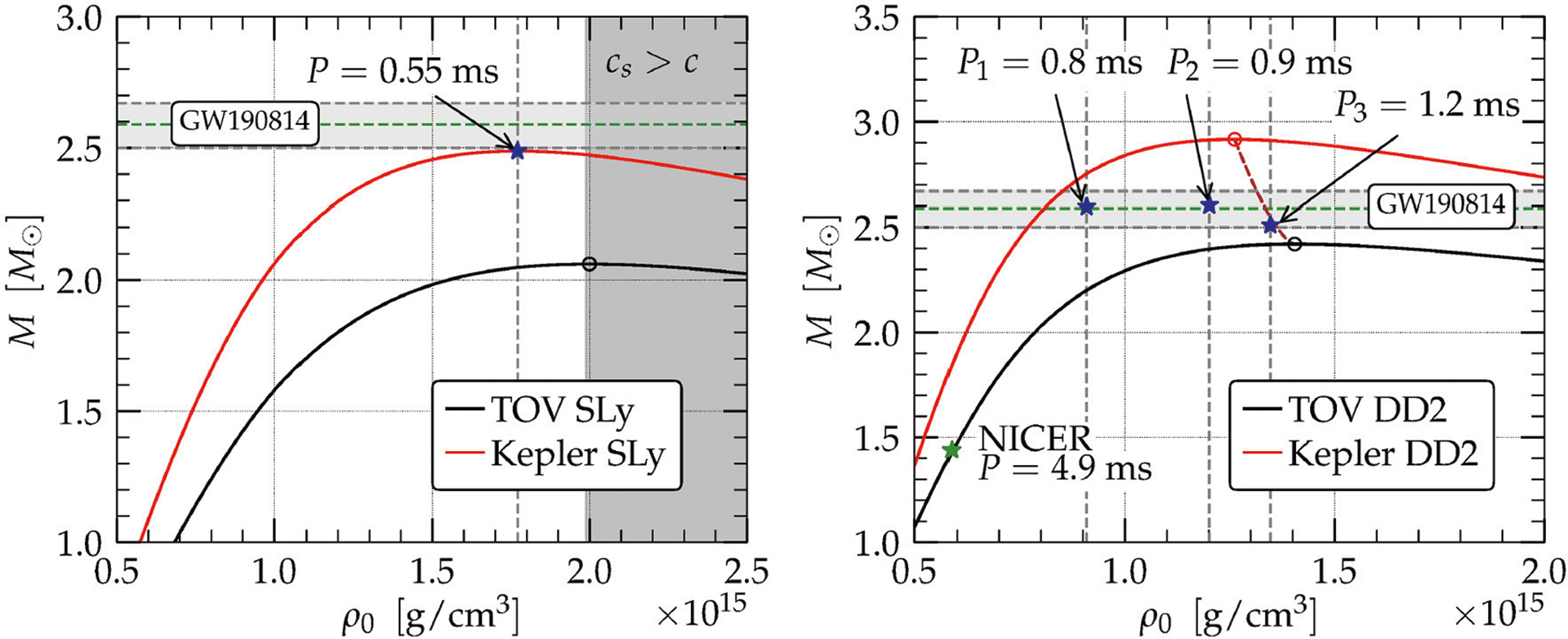
Two possibilities for the EOS of a NS companion in GW190814. The scenario on the left, which employs the SLy (soft) EOS, fails to provide a model for a uniformly rotating star, even at maximum uniform rotation. On the contrary, the scenario on the right that employs the DD2 (stiff) EOS succeeds and demonstrates the possibility of a slowly rotating NS. The lower (black) curves represent spherical, non-rotating models, while the upper (red) curves represent uniformly rotation models spinning at the Keplerian (mass-shedding) limit (from Tsokaros et al., 2020a).

One of the most intriguing GW detections to date was event GW190814 (Abbott et al., 2020a), a binary coalescence whose primary component had mass $m_{1}=23.2_{-1.0}^{+1.1}M_{⊙}$ and therefore is a BH, while the secondary had mass $m_{2}=2.59_{-0.09}^{+0.08}M_{⊙}$, placing it at the boundary of the so-called “mass gap” and making its identification uncertain. Further ambiguity was added by the absence of an EM counterpart. While the nature of this compact object is not yet known, it was already suggested by Abbott et al. (2020a) that it can be a rapidly rotating NS, whose dimensionless spin was estimated to be 0.49 ≲ *a*_NS_/*M*_NS_ ≲ 0.68 (Most et al., 2020). For this scenario to be viable the maximum mass of a spherical, non-rotating cold NS has to be ≳ 2.1 *M*_⊙_ (Most et al., 2020; Tsokaros et al., 2020a). Requiring rapid rotation for a NS companion in GW190814 is a direct consequence of the likely upper limits (2.2 − 2.3 *M*_⊙_) placed on a spherical, non-rotating NS mass by event GW170817 (Margalit and Metzger, 2017; Shibata et al., 2017, 2019; Rezzolla et al., 2018; Ruiz et al., 2018a). These upper limits were mostly based on the assumption that the companions in GW170817 were slowly rotating. Assuming rapid uniform NS rotation, instead, the upper limit allowed by event GW170817 increases (Abbott et al., 2017c; Ruiz et al., 2018a) and can explain the 2.6 *M*_⊙_ compact object in GW190814 as a slowly rotating NS. In fact, by allowing for the uncertainties and adopting a sufficiently stiff EOS, even a non-rotating NS can explain GW190814 (Tsokaros et al., 2020a). Note that although no robust discovery of a BHNS exists yet, the NSs in the 20 known NSNS systems (Tauris et al., 2017; Zhu et al., 2018) have low dimensionless spins. While one cannot draw definitive conclusions from these limited number of observations, one might safely argue that if spin-down due to EM emission is as efficient as in currently known binaries, then any scenario involving a highly spinning NS either in an NSNS (like GW170817) or in an BHNS system (like GW190814) is not probable. In summary, invoking rotation to explain the companion to the BH object in GW190814 depends on the stiffness of the EOS and the assumptions of the maximum mass of a spherical NS. For a soft EOS (low spherical maximum mass) such as SLy (Douchin and Haensel, 2001) rapid rotation is not sufficient, while for sufficiently stiff EOS such as DD2 (Hempel and Schaffner-Bielich, 2010) rapid rotation may not even be necessary. Such EOSs are neither rejected nor favored by GW170817, and they are in accordance with the results of NICER (see Figure 4).

## 3. NSNS MERGERS: REMNANTS AND INCIPIENT JETS

Numerical simulations of NSNS binaries are somewhat simpler than BHNS binaries, since the latter must treat the BH singularity. Some of the first numerical studies of NSNSs employed Newtonian gravity, modeling the NS as a polytrope (Gilden and Shapiro, 1984; Oohara and Nakamura, 1989; Rasio and Shapiro, 1992, 1994; Shibata et al., 1992, 1993; Xing et al., 1994; New and Tohline, 1997). For circular orbit binaries it was found that following the binary merger, a highly differentially rotating remnant is formed. However, their simulations could not track its possible collapse to a BH with Newtonian gravity. Motivated by models of sGRBs and the ejection of r-process nuclei, Davies et al. (1994), Ruffert et al. (1996), and Ruffert and Janka (1998) extended the previous results by incorporating a simple treatment of the nuclear physics in their numerical calculations. One of the first approaches used to simulate NSNS coalescence in GR was the “conformal flatness approximation” (CFA) used by Wilson and Mathews (1995), which has been followed by several other treatments with increasing sophistication. Oechslin et al. (2002) evolved NSNS binaries using a Lagrangian SPH code with a multigrid elliptic solver to handle the gravitational field equations and corotating initial configurations. Faber et al. (2004) subsequently performed SPH simulations in the CFA using a spectral elliptic solver in spherical coordinates and employed the quasi-equilibrium, irrotational binary models of Taniguchi and Gourgoulhon (2002). These models are constructed using the conformal thin-sandwich formalism (York, 1999). Oechslin et al. (2007) extended their earlier studies by including the influence of a realistic nuclear EOS. These simulations showed that the dynamics and the final outcome of the merger depend sensitively on the EOS and the binary parameters, such as the gravitational mass of the system and its mass ratio. The first fully GR simulations of NSNS undergoing merger were performed by Shibata and Uryu (2000), Shibata and Uryū (2002), and Shibata et al. (2003) using a polytropic EOS to model the stars. Since then, great progress has been made to model NSNSs incorporating realistic microphysics and magnetic field effects in full GR and in alternative theories of gravity. In the following we only review full GR studies of these binaries. For earlier reviews and references, see, e.g., Baumgarte and Shapiro (2010) and Shibata (2015).

### 3.1. Nonmagnetized Evolutions

One of the first questions numerical studies of NSNS mergers in full GR were compelled to address was under what conditions the highly differentially rotating star remnant collapses to a BH. The uncertainties in the nuclear EOS, combined with theoretical arguments invoking GW170817 and its EM counterparts, allow non-rotating NSs with a maximum mass limit in the range $M_{\text{max}}^{\text{sph}}\sim 2.1-2.4M_{⊙}$ (Margalit and Metzger, 2017; Shibata et al., 2017, 2019; Rezzolla et al., 2018; Ruiz et al., 2018a). Uniform rotation allows NSs with up to ~ 20% more mass (“supramassive stars”; as coined by Cook et al., 1994a,b). Even larger masses can be supported against collapse with centrifugal support if the star is differentially rotating. Such stars were first constructed and explored by Baumgarte et al. (2000), who built dynamically stable Γ = 2 polytropic models with masses ≳ 3 − 4 *M*_⊙_. They coined the label “hypermassive neutron star” (HMNS) to describe such stars. It was demonstrated by Duez et al. (2004) that shear viscosity drives a HMNS to collapse to a BH on a (secular) viscous timescale and by Duez et al. (2006) that turbulent magnetic viscosity induced by MRI can also drive the secular collapse of the latter magnetic HMNSs. These viscous effects compete with neutrino and GW emission (when the HMNS remnant is non-axisymmetric) to drive collapse. In NSNS binaries, the fate of the remnant depends on the total mass of the NSNS binary, as we shall now discuss.

Shibata and Uryu (2000) and Shibata and Taniguchi (2006) found that there is a threshold mass *M*_th_ above which the remnant collapses immediately on a dynamical timescale to a BH, independently of the initial binary mass ratio. This threshold value depends strongly on the EOS. For Γ = 2 polytropes $M_{\text{th}}\approx 1.7M_{\text{max}}^{\text{sph}}$, while for stiffer EOSs, such as APR (Akmal et al., 1998) and SLy (Douchin and Haensel, 2001), it is $\sim 1.3-1.35M_{\text{max}}^{\text{sph}}$. Shibata and Taniguchi (2006) also found that in the case of “prompt” collapse to a BH, the mass of the disk remnant increases sharply with increasing mass ratio for a fixed gravitational mass and EOS. In addition, if the mass of the binary is less than *M*_th_ the disk remnant turns out to be more massive than for those whose mass is larger than *M*_th_. For binaries with *M* < *M*_th_ their remnants form a transient, highly deformed HMNS which, after ~ 8–50 ms, undergoes a “delayed” collapse to a BH surrounded by a significant accretion disk. The collapse occurs due to angular momentum losses from gravitational radiation in these simulations where neutrino cooling and magnetic fields are absent (Baiotti et al., 2008; Kiuchi et al., 2009; Rezzolla et al., 2010; Dietrich et al., 2015; Ruiz et al., 2019). These results have been extended by Hotokezaka et al. (2011) using a piecewise polytropic representation of nuclear EOSs (Özel and Psaltis, 2009; Read et al., 2009). It was found that the threshold value is in the range $1.3≲M_{\text{th}}/M_{\text{max}}^{\text{sph}}≲1.7$. These results were confirmed also for realistic finite-temperature EOSs (Bauswein et al., 2013). In addition, the ratio between the threshold mass and maximum mass is tightly correlated with the compactness of the $M_{\text{max}}^{\text{sph}}$. Finally, less massive binary mergers form a dynamically stable NS remnant that may collapse on longer time scales once dissipative processes, such as neutrino dissipation or gravitational radiation, take place (Cook et al., 1994a,b; Lasota et al., 1996; Breu and Rezzolla, 2016).

Most of the numerical calculations to date have focused on quasi-circular irrotational binaries, though it is expected that spin can modify the threshold value of prompt collapse, or at least change the lifetime of the remnant. Preliminary results reported by Kastaun and Galeazzi (2015), Dietrich et al. (2017), Ruiz et al. (2019), and Chaurasia et al. (2020) showed that depending on the NS spin, the lifetime of the remnant may change from ~ 8 to ≳ 40 ms. Effects of NS spin on the inspiral have been explored by Kiuchi et al. (2017), Bernuzzi et al. (2014), Dietrich et al. (2018), and Tsokaros et al. (2019a). On the other hand, the dynamically captured NSNS mergers that may arise in dense stellar regions, such as globular clusters, have been studied by East and Pretorius (2012). These results showed that *M*_th_ and the mass of the disk remnant depend not only on the EOS but also on the impact parameter. The calculations by Paschalidis et al. (2015) and East et al. (2016) demonstrated that the HMNS formed through dynamical capture may undergo the one-arm non-axisymmetric (mode *m* = 1) instability.

During merger, shock heating produces temperatures as high as ~ 100 MeV at the contact layer between the two stars. Subsequent compressions lead to average-temperatures of the order of 10 MeV in the central core of the NSNS remnant (Bauswein et al., 2010), and hence the binary remnant can be a strong emitter of neutrinos. The timescale of neutrino cooling radiation (typically ≲ 1 s) may also strongly affect the HMNS lifetime (Sekiguchi et al., 2011). Effects of neutrino cooling on the dynamical ejecta that can give rise to observable kilonova signatures have been studied in Radice et al. (2016), Lehner et al. (2016), and Sekiguchi et al. (2015) (see Radice et al., 2020 for a recently review). It is worth noting that the calculations of Bauswein et al. (2010) and Just et al. (2016) show that neutrino heating drives a wind from the surface of the remnant, creating very baryon-loaded environments in the polar regions that prevent the formation of incipient jets. Therefore, MHD processes are likely be a key ingredient to overcome this and to trigger the formation of relativistic jets.

**FIGURE 5 |:**
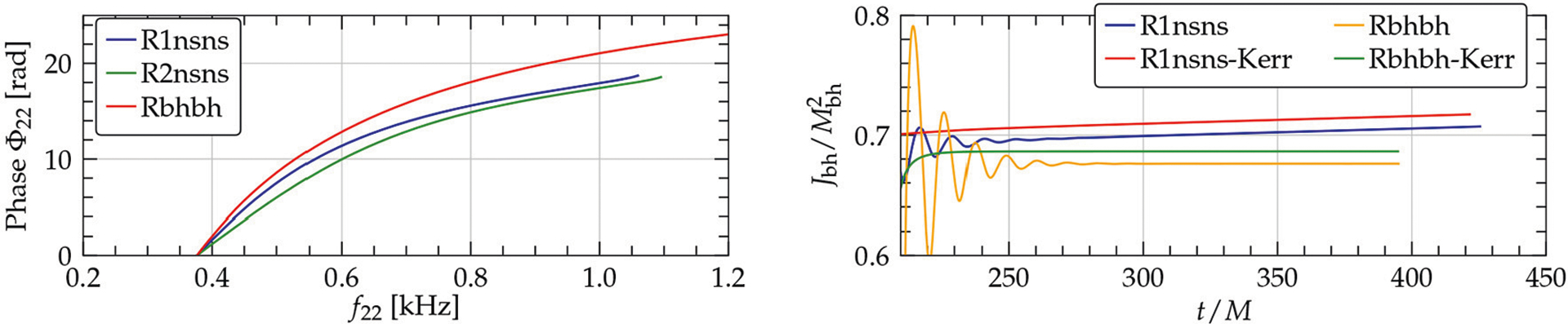
**(Left)** GW phase vs. frequency for the NSNS binary using two resolutions (R1nsns,R2nsnsn) and a BHBH binary having the same gravitational mass. **(Right**) Dimensionless spin of the remnant BH for the NSNS (R1nsnsn) and the BHBH (Rbhbh) binary. Also shown is the dimensionless spin as computed from the Kerr formula for the two systems (from Tsokaros et al., 2020c).

Numerical simulations of NSs having a mass that falls inside the so-called “mass-gap” are scarce. The first such simulation was performed by Tsokaros et al. (2020c) with a binary NSNS in a quasi-equilibrium circular orbit. The gravitational mass of the binary was *M* = 7.90 *M*_⊙_, and each star is identical and has a compactness of C=0.336. This value, which is even higher than the maximum possible compactness that can be achieved by solitonic boson stars (Palenzuela et al., 2017), is slightly smaller than the limiting compactness $C_{\text{max}}=0.355$ set by causality (Lattimer and Prakash, 2016). To build these binaries, Tsokaros et al. (2020c) employed the ALF2 EOS (Alford et al., 2005), but replaced the region where the rest-mass density satisfies *ρ*_0_ ≥ *ρ*_0,*s*_ = *ρ*_0,nuc_ = 2.7×10^14^ gr/cm^3^ by the maximally stiff EOS

(8) $$P=\rho -\rho_{s}+P_{s},$$

with sound speed equal to the speed of light. Here *ρ* is the total energy density, and *P*_*s*_ the pressure at *ρ*_*s*_, assumed known. The quasi-equilibrium initial data were built using the COCAL code (see e.g., Tsokaros et al., 2015, 2016). Due to the large compactions of the NSs the binary stars exhibit no tidal disruption up until merger, whereupon a prompt collapse is initiated even before a common core forms. Within the accuracy of the simulations the BH remnant from this NSNS binary exhibits ringdown radiation that is not easily distinguishable from a perturbed Kerr BH. Right panel of Figure 5 displays the dimensionless spin from the BH remnant from the NSNS and that from a BHBH binary having the same gravitational (ADM) mass. Also shown are the remnant spins as computed from the analytic Kerr formula whose input is the ratio *L*_*p*_/*L*_*e*_ of the polar to equatorial circumference. However, the inspiral leads to phase differences of the order of ~ 5 rad (left panel of Figure 5) over an ~ 81 km separation (~ 1.7 orbits). Although such a difference can be measured by current GW laser interferometers (e.g., LV scientific collaboration observatories), uncertainties in the individual masses and spins will likely prevent distinguishing such compact, massive NSNSs from BHBHs.

### 3.2. Magnetized Evolutions

Although NS may have very large magnetic fields (≳ 10^14^ G) at birth, it is expected that cooling processes significantly reduce their magnitudes (Pons et al., 2009). Pulsar observations indicate that the characteristic surface magnetic field strength of NSs is ~ 10^10^ − 10^12^ G (Lorimer, 2008; Lyne and Graham-Smith, 2012; Miller et al., 2019; Semena et al., 2019). Nevertheless, magnetic instabilities such as the Kelvin-Helmholtz instability (KHI; see e.g., Price and Rosswog, 2006; Anderson et al., 2008; Kiuchi et al., 2015a,b), MRI (see e.g., Duez et al., 2006; Shibata et al., 2006; Siegel et al., 2013; Kiuchi et al., 2015b), and magnetic winding (see e.g., Baumgarte et al., 2000; Kiuchi et al., 2015a; Sun et al., 2019) triggered during and after the NSNS merger can substantially boost the strength of these weak fields.

**FIGURE 6 |:**
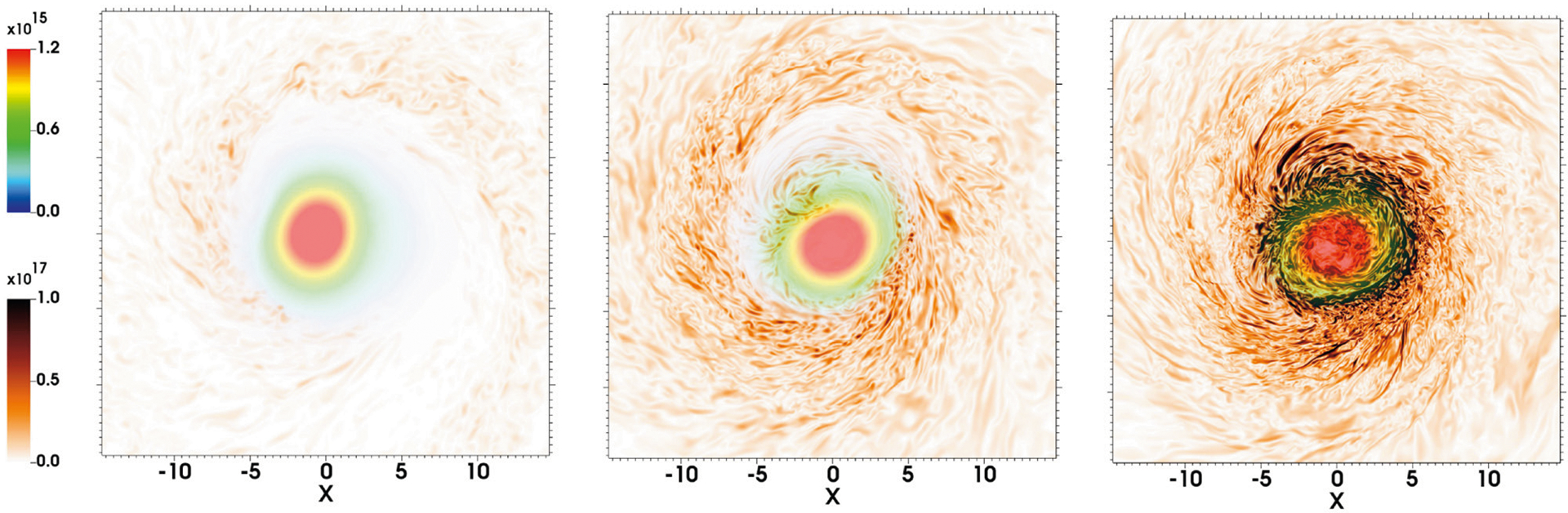
NS rest-mass density *ρ*_0_ (upper left rainbow colorbar) and magnetic field (lower left brownish colorbar) on the orbital plane at *t* = 10.0 ms following an NSNS merger at three different resolutions [Δ*x* = 147 m **(left)**, at Δ*x* = 74 m **(middle)**, and Δ*x* = 37 m **(right)**]. The initial magnetic field strength is 5 × 10^11^ G (adapted from Aguilera-Miret et al., 2020).

High-resolution simulations are required to properly capture the above instabilities because their fastest growing modes have short wavelengths. Kiuchi et al. (2015a,b) systematically studied the effects of numerical resolution on the magnetic field amplification in NSNS mergers and found that, at the unprecedented resolution of Δ*x* = 17.5 m, an initial magnetic field of 10^13^ G is amplified to values ≳ 10^15^ G in the bulk of the remnant, with local values peaking at ~ 10^17^ G, after 5 ms following merger. Recently, the calculations by Aguilera-Miret et al. (2020) reported that at a resolution of Δ*x* = 37 m an initial magnetic field of 5 × 10^11^ G is amplified to values of ~ 10^17^ G after about 10 ms following merger (see Figure 6). These extremely high-resolution simulations are computationally quite expensive and currently inaccessible for general studies. Typical NSNS simulations use a resolution ≳ 120 m (see e.g., Ciolfi et al., 2019; Ruiz et al., 2019; Bernuzzi et al., 2020; Vincent et al., 2020; Weih et al., 2020). To overcome the lack of resolution, some works have adopted subgrid models to mimic the effect of magnetic instabilities (see e.g., Giacomazzo et al., 2015; Palenzuela et al., 2015; Aguilera-Miret et al., 2020; Radice, 2020), while others have employed high, but dynamically weak initial magnetic fields to mimic the resulting magnetic field following the merger (see e.g., Ruiz et al., 2016; Ciolfi et al., 2019; Mösta et al., 2020). These two approaches allow the tracking of the secular evolution of the a quasi-stationary NSNS remnant consisting of a HMNS that ultimately undergoes delayed collapse to a highly spinning BH surrounded by an accretion disk with a strong magnetic field with finite computational resources.

Some of the first long-term ideal GRMHD studies of NSNS mergers were performed by Anderson et al. (2008) and Liu et al. (2008) using Γ = 2 polytropes endowed with a 10^16^ G polodial magnetic field confined to the NS interior (see Equation 5). The simulations of Anderson et al. (2008) reported the formation of a long-lived HMNS. During this phase, turbulent magnetic fields transport angular momentum away from the center, inducing the formation an axisymmetric central core that eventually collapses to a spinning BH. Liu et al. (2008) reported the evolution of equal and unequal binaries that promptly collapse to a BH following merger, surrounded by a disk with ≲ 2% of the total rest mass of the binary. Neither an outflow nor a magnetic field collimation were found.

**FIGURE 7 |:**
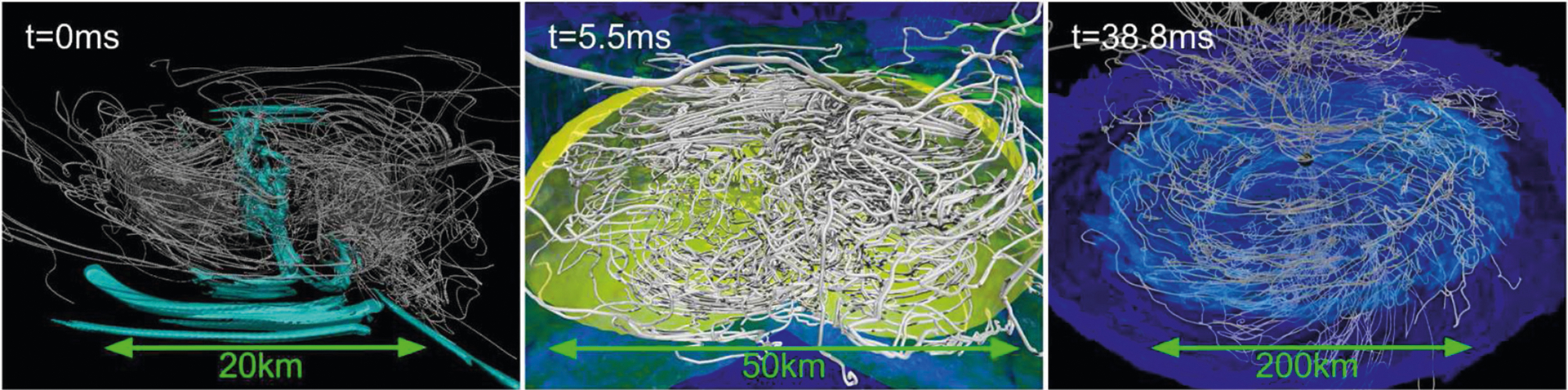
NS rest-mass density and magnetic field lines at *t* − *t*_mrg_ ≈ 0.0 ms **(left)**, *t* − *t*_mrg_ ≈ 5.5 ms **(middle)**, and *t* − *t*_mrg_ ≈ 38.8 ms **(right)** following a NSNS merger. Here *t*_mrg_ is the merger time. Cyan color on the left panel displays magnetic fields stronger than 10^15.6^ G. Yellow, green, and dark blue colors on the middle panel show rest-mass densities of 10^14^, 10^12^, and 10^10^ g/cm^3^, respectively. Light and dark blue colors on the right panel indicate rest-mass densities of 10^10.5^, and 10^10^ g/cm^3^, respectively (from Kiuchi et al., 2015b).

The calculations of Rezzolla et al. (2011) reported that ~ 12 ms after the collapse of a HMNS remnant, MHD instabilities develop and form a central, low-density, poloidal-field funnel, though there were no evidences of an outflow. The initial data consist of a binary polytrope initially endowed with a 10^12^ G poloidal magnetic field confined to the stellar interior. The highest resolution used in these studies was Δ *x* ≈ 221 m. A subsequent high-resolution study by Kiuchi et al. (2015b), employing an H4 EOS (Glendenning and Moszkowski, 1991) with seed poloidal magnetic fields confined to the stellar interior, found that during merger, the magnetic field is steeply amplified due to the KHI. In their high-resolution case (Δ*x* = 70 m) the amplification is 40–50 times larger than that in the low-resolution case (Δ*x* = 150 m). In contrast to the results of Rezzolla et al. (2011), the ram pressure of the fall-back debris prevents the formation of a coherent poloidal field. As the frozen-in magnetic field lines are anchored to the fluid elements, an outflow, which was not seen after 40 ms following merger (see Figure 7), is presumably necessary to generate a coherent poloidal magnetic field.

**FIGURE 8 |:**
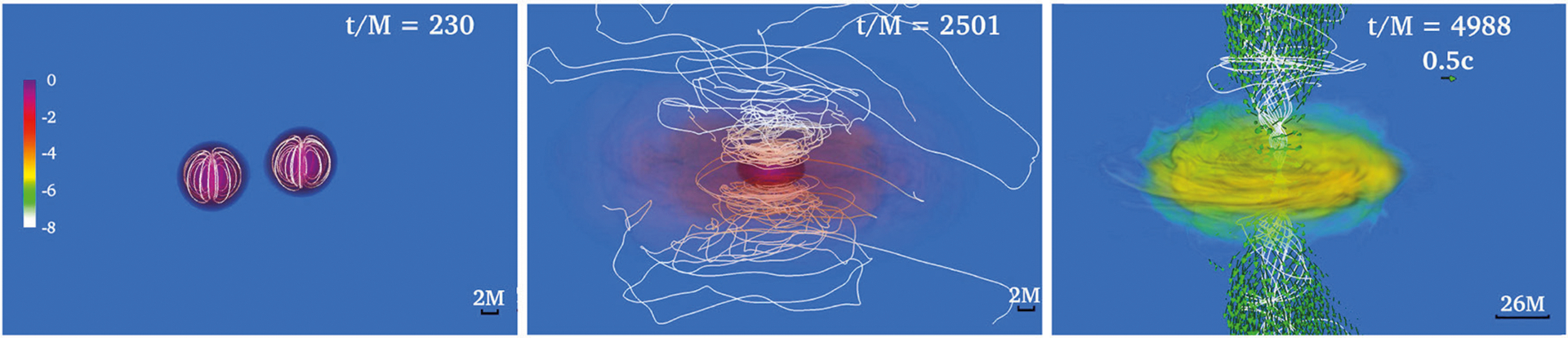
NS rest-mass density *ρ*_0_ normalized to its initial maximum value *ρ*_0,max_ = 5.9 × 10^14^(1.625 *M*_⊙_/*M*_NS_)^2^ g/cm^3^ (log scale) at selected times for an NSNS merger. Arrows display plasma velocities and white lines show magnetic field lines. Here *M* = 1.47 × 10^−2^ (*M*_NS_/1.625*M*_⊙_) ms = 4.43(*M*_NS_/1.625*M*_⊙_) km (Snapshots from case IH in Ruiz et al., 2016).

Ruiz et al. (2016) evolved the same NSNS configuration as in Rezzolla et al. (2011) but using higher resolution (Δ*x* = 152 m). As this resolution is still not enough to properly capture the growth of the magnetic field due to the KHI, Ruiz et al. (2016) endowed the initial NSs with dynamically weak, purely poloidal magnetic fields with strengths *B*_pole_ ≃1.75×10^15^(1.625*M*_⊙_/*M*_NS_) G at the poles of the stars, which matches the values of the field strength in the HMNS reached in Kiuchi et al. (2015b). It was found that by ~ 4, 000M ~ 60(*M*_NS_/1.625*M*_⊙_) ms following BH formation, the magnetic field above the BH poles has been wound into a tight, helical funnel inside of which fluid elements begin to flow outward: this is an incipient jet (see Figure 8). The lack of a jet in Kiuchi et al. (2015b) can be attributed to the persistent fall-back debris in the atmosphere, which increases the ram pressure above the BH poles. Therefore, a longer simulation like the one in Ruiz et al. (2016) is required for jet launching. Notice that jet launching may not be possible for all EOSs if the matter fall-back timescale is longer than the disk accretion timescale (Paschalidis, 2017).

**FIGURE 9 |:**
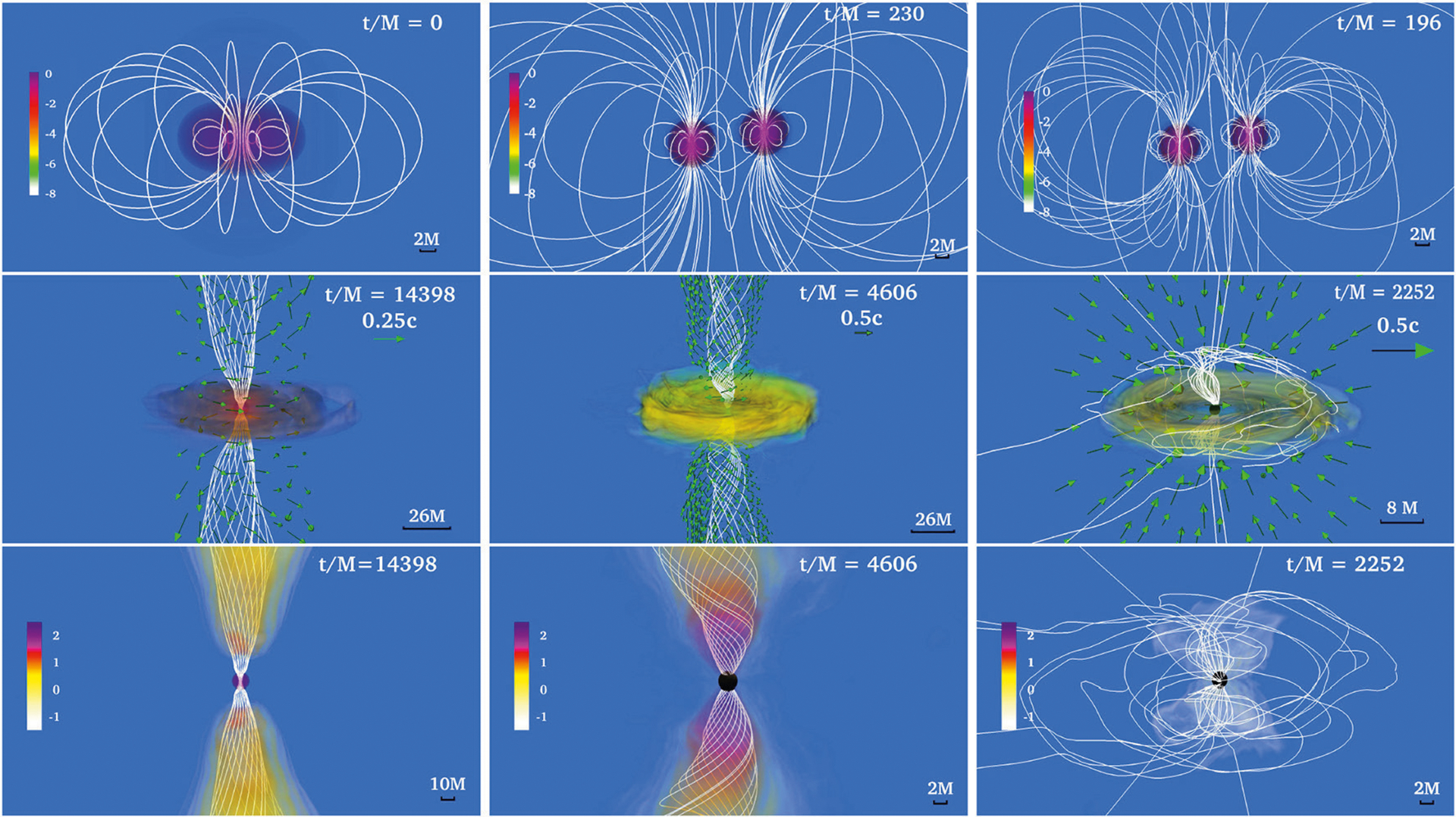
NS rest-mass density *ρ*_0_ normalized to its initial maximum value (log scale) for a NSNS binary that forms: a stable, supramassive remnant (**left**); a HMNS remnant that undergoes delayed collapse (**middle**); and a remnant that undergoes prompt collapse (**right**). Top row displays the NSs at the time of magnetic field insertion, while middle row displays the outcome once the remnant has reached quasi-equilibrium. Bottom row shows the force-free parameter *B*^2^/(8*πρ*_0_) (log scale). White lines represent magnetic field lines, while arrows represent fluid velocity flow vectors. The field lines form a tightly wound helical funnel and drive a jet following delayed collapse, but not in the other two cases. Here *M* = 0.0136(*M*_tot_/2.74*M*_⊙_)ms = 4.07(*M*_tot_/2.74*M*_⊙_) km; therefore quasi-equilibrium for the supramassive case (left column) is achieved at *t* ~ 200 ms (from Ruiz et al., 2018a).

In addition, Ruiz et al. (2016) studied the impact of the magnetic configuration on the jet launching time. For this the NSs were endowed with the pulsar-like interior + exterior magnetic field generated by the vector field in Equation (6). To reliable evolve the exterior magnetic field, Ruiz et al. (2016) adopted the atmosphere treatment previously used by Paschalidis et al. (2015). As illustrated in Figure 8, a magnetically-driven jet is launched on the same time scale (see second column in Figure 9). Unlike in the BHNS case in Paschalidis et al. (2015), where the magnetic field grows following BH formation, the MRI and magnetic winding in the HMNS already amplifies the magnetic field to saturation levels before the onset of collapse to a BH. The incipient jet is then launched by the BH + disk remnant due to the emptying of the funnel as matter accretes onto the BH, thereby driving the magnetic field regions above the BH poles to nearly force-free values (*B*^2^/8*πρ*_0_ ≫ 1). Notice that the initial magnetic field configuration affects the level of collimation of the incipient jet. The opening half-angle of the pulsar-like magnetic field case is ~ 25°, while for the magnetic field confined to the stellar interior it is ~ 30°. The Lorentz factor in the outflow is Γ_L_ ~ 1.2. Thus, the incipient jet is only mildly relativistic. However, the force-free parameter inside the funnel is *B*^2^/8*πρ*_0_ ~ 100 (see bottom panel of the second column in Figure 9), and therefore fluid elements can be accelerated to Γ_L_ ~ 100 (Vlahakis and Konigl, 2003). The lifetime of the accretion disk (jet’s fuel) is ~ 100(*M*_NS_/1.625*M*_⊙_) ms and hence consistent with sGRB lifetimes (Bhat et al., 2016; Lien et al., 2016; Svinkin et al., 2016; Ajello et al., 2019). The outgoing Poynting luminosity is *L*_*EM*_ ~ 10^50.3^ −10^51.3^ erg/s, roughly consistent with the luminosity expected from the BZ effect (see Equation 7) and the universal merger model (Shapiro, 2017). As this equation is strictly valid for highly force-free magnetospheres, it is likely that any deviation from the expected Poynting luminosity is due to partial baryon-loaded surroundings.

To further assess the robustness of the emergence of the incipient jet in NSNS mergers, numerical studies by Ruiz et al. (2019, 2020b) probed the impact of the NS spin and the orientation of the seed poloidal magnetic field on the formation and lifetime of the HMNS, BH + disk remnant, and the jet launching time. Ruiz et al. (2019) found that the larger the co-rotating NS spin, the more massive the accretion disk, and hence the longer the jet’s lifetime. In addition, the larger the NS spin, the shorter the time delay between the peak GW and the emergence of the incipient jet. On the other hand, the simulations of Ruiz et al. (2020b) suggest that there is a threshold value of the inclination of magnetic dipole moment with respect to the orbital angular momentum $\vec{L}$ of the binary beyond which jet launching is suppressed. A jet is launched whenever a net poloidal magnetic flux with a consistent sign along $\vec{L}$ is accreted onto the BH once *B*^2^/8*πρ*_0_ ≫ 1 above the BH poles. Tilted magnetic fields change the magnitude of this poloidal field component.

**FIGURE 10 |:**
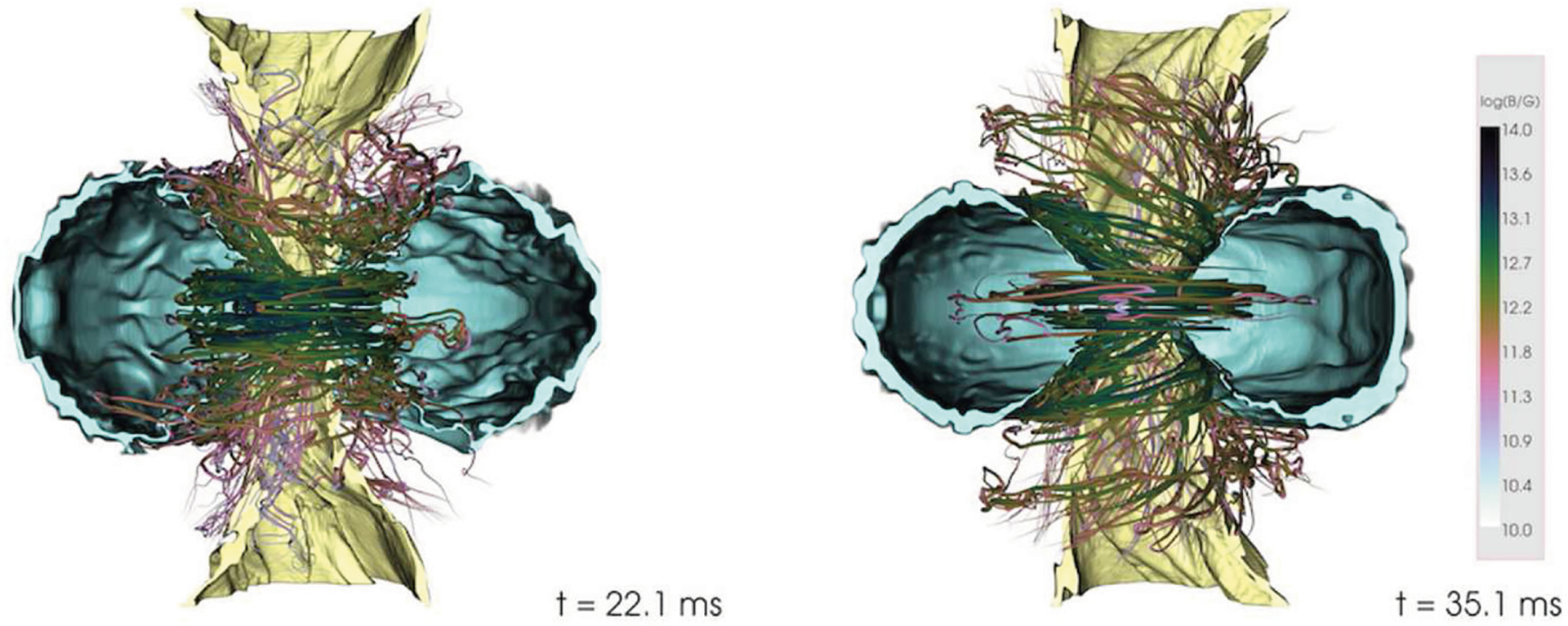
Magnetic field lines at ~ 22 ms **(left)** and ~32 ms **(right)** following an NSNS merger, along with two isosurfaces of rest-mass density 10^8^ (yellow) and 10^10^ g/cm^3^ (cyan), cut off for *y* < 0 (from Kawamura et al., 2016).

Kawamura et al. (2016) and Ciolfi et al. (2017) probed the effects of different EOSs, different mass ratios, and different orientations of poloidal magnetic field confined to the NS interior, with strengths ~ 10^12^ − 10^15^ G. The NSNS binaries were evolved with a resolution Δ*x* ≳ 177 m. These calculations found that after 22 ms following merger, an organized magnetic field structure above the BH emerges, though magnetically-driven outflow was not observed (see Figure 10). The lack of an incipient jet is likely due to insufficient resolution to properly capture the magnetic instabilities that boost the magnetic field strength to ≳ 10^15.5^G, an essential ingredient for jet launching, and/or to too short evolutions times. Notice that the ram-pressure of the fall-back debris depends strongly on the EOS. More baryon-loaded surroundings require stronger magnetic fields to overcome the ram-pressure, delaying the launch of the jet while the fields amplify.

The previous numerical studies involved NSNS mergers leading to the formation of a transient HMNS undergoing delayed collapse to a BH. The possibility of jet launching from a stable supramassive NS remnant has recently been investigated by Ruiz et al. (2018a), Ciolfi et al. (2019), and Ciolfi (2020). The calculation of Ruiz et al. (2018a) reported a long-term (~ 200 ms) simulation of a supramassive NS remnant initially threaded by a pulsar-like magnetic field. It was found that magnetic winding induces the formation of a tightly-wound-magnetic-field funnel within which some matter begins to flow outward (see first column in Figure 9). The maximum Lorentz factor in the outflow is Γ_L_ ~ 1.03, and the force-free parameter inside the funnel is *B*^2^/8*πρ*_0_ ≪ 1. The Poynting luminosity is ~ 10^43^ erg/s, and roughly matches the GR pulsar spindown luminosity (Ruiz et al., 2014). These calculations suggest that a supramassive NS remnant probably cannot be the progenitor of a sGRB. This has been confirmed by the simulations of Ciolfi et al. (2019) and Ciolfi (2020), which reported the emergence of an outflow with a maximum Lorentz factor of Γ_L_ ≲ 1.05 after ≳ 212 ms following the merger of a magnetized, low-mass NSNS. Recently, the calculation of Mösta et al. (2020) suggested that neutrino effects may help reduce the baryon-load in the region above the poles of the NS, inducing a growth of the force-free parameter in the funnel. They found a maximum Lorentz factor of Γ_L_ ≲ 5.0 inside the funnel. Thus, neutrinos processes may help to trigger the launching of an incipient jet. Finally, the numerical simulations of Ruiz and Shapiro (2017), who did not include neutrinos, probed whether or not prompt collapse NSNS remnants (BHs with small accretion disks) can launch incipient jets. No evidence of an outflow or magnetic field collimation was found (see third column on Figure 9). It was argued that the KHI and MRI do not have enough time to amplify the magnetic field prior to BH formation, and hence a jet can not be launched.

Although supramassive NS or prompt collapse remnants may not launch magnetically-driven jets, they may be the progenitors of fast radio bursts (FRBs)—a new class of radio transients lasting less than a few tens of milliseconds (Lorimer et al., 2007; Thornton et al., 2013). Falcke and Rezzolla (2014) have suggested that magnetic field reconfigurations during the collapse of a supramassive NS can induce a burst of EM radiation consistent with that of typical FRBs. Palenzuela et al. (2013) studied EM counterparts from the inspiral and merger of a NSNS binaries using full GR resistive MHD simulations. They found that the interaction between the stellar magnetospheres extracts kinetic energy from the binary and powers radiative Poynting fluxes as large as *L*_EM_ ≃ 10^41−44^(*B*/10^12^*G*)^2^ erg/s in a few milliseconds. Motivated by these results, Paschalidis and Ruiz (2018) performed numerical simulations of prompt collapse NSNS mergers in which the NSs are initially endowed with a pulsar-like magnetic field. Combining their numerical results with population studies, they concluded that FRBs may be the most likely EM counterpart of prompt collapse NSNSs, as previously claimed by Totani (2013).

### 3.3. GW170817 and the NS Maximum Mass

Event GW170817 (Abbott et al., 2017c) marked not only the first direct detection of a NSNS binary undergoing merger via GWs but also the simultaneous detection of the sGRB GRB 170817A, and kilonova AT 2017gfo, the latter with its afterglow radiation in the radio, optical/IR, and X-ray bands (Kozlova et al., 2017; von Kienlin et al., 2017). These observations have been used to impose constraints on the physical properties of a NS, and in particular, on the maximum mass of a non-rotating spherical NS, $M_{\text{max}}^{\text{sph}}$.

Margalit and Metzger (2017) argued that following the merger of the NSNS progenitor of GW170817, a transient HMNS is formed which collapses to a BH on a timescale of ~ 10–100 ms, producing the observed kilonova ejecta expanding at mildly relativistic velocities. This conclusion combined with the GW observation, led to their tight prediction that $M_{\text{max}}^{\text{sph}}≲2.17M_{⊙}$ with 90% confidence. On the other hand, Shibata et al. (2017) summarized a number of their relativistic hydrodynamic simulations favoring a long-lived, massive NS remnant surrounded by a torus to support their inferred requirement of a strong neutrino emitter that has a sufficiently high electron fraction (*Y*_*e*_ ≳ 0.25) to avoid an enhancement of the ejecta opacity. This argument led then to the results that $M_{\text{max}}^{\text{sph}}\sim 2.15-2.25M_{⊙}$. A recently review of these calculations by Shibata et al. (2019) using energy and angular momentum conservation laws again lead to $M_{\text{max}}^{\text{sph}}≲2.3M_{⊙}$. Rezzolla et al. (2018) assumed that the transient GW170817 remnant collapsed to a spinning BH once it had reached a mass close to but below the maximum mass of a supramassive star. This assumption combined with their quasi-universal rotating NS model relations led to $M_{\text{max}}^{\text{sph}}≲2.16_{-0.15}^{+0.17}M_{⊙}$. Ruiz et al. (2018a) used the existence of the sGRB GRB170817A, combined with their conclusion that only a NSNS merger that forms an HMNS that undergoes delayed collapse to a BH can be the progenitor of an engine that powers an sGRB (see Figure 9), to impose the bound $M_{\text{max}}^{\text{sph}}≲2.74/\beta$ (for low spin priors), where *β* is s the ratio of the maximum mass of an uniformly rotating NS (supramassive limit) to the maximum mass of a non-rotating star. Causality arguments allow *β* to be as high as 1.27, while most realistic candidate EOSs predict *β* ≃ 1.2, yielding $M_{\text{max}}^{\text{sph}}$ in the range ~ 2.16–2.28*M*_⊙_. If instead one assumes high spin priors in interpreting the data for GW170817 their maximum mass limit becomes ~ 2.22–2.35*M*_⊙_. Thus, the different analyses seem to converge on a value for $M_{\text{max}}^{\text{sph}}\sim 2.2-2.3M_{⊙}$.

## 4. ERGOSTARS: POTENTIAL MULTIMESSENGER ENGINES

In the previous two sections, we summarized GRMHD simulations showing that the key requirement for the emergence of a magnetically-driven jet is the existence of a spinning BH remnant surrounded by an appreciable disk. In addition, these simulations also suggest that the BZ process is the driving mechanism to power them.

The BZ process can be explained using the membrane paradigm (Thorne et al., 1986), in which the BH horizon is treated as a spherical, rotating conductor of finite resistivity. The magnetic field lines threading the BH horizon transfer rotational kinetic energy from a spinning BH to an outgoing Poynting and matter flux. However, Komissarov (2002, 2004, 2005) has argued that the BH horizon is not the “driving force” behind the BZ mechanism, but rather it is the ergoregion. To disentangle the effects of the BH horizon and the ergoregion, Ruiz et al. (2012) performed force-free, numerical evolutions of magnetic fields on the *fixed* matter + metric background of an “ergostar” (a star with an internal ergoregion but no horizon) modeled by the EOS of incompressible, homogeneous matter with constant total mass-energy density. In addition, the same magnetic fields were evolved on the fixed background of a spinning BH. Ruiz et al. (2012) found that once the system reaches quasiequilibrium, the configuration of the EM fields and currents on both backgrounds are the same, in agreement with Komissarov (2002, 2004, 2005). These preliminary results suggest that the BZ process is a mechanism driven by the ergoregion, and not by the BH horizon.

**FIGURE 11 |:**
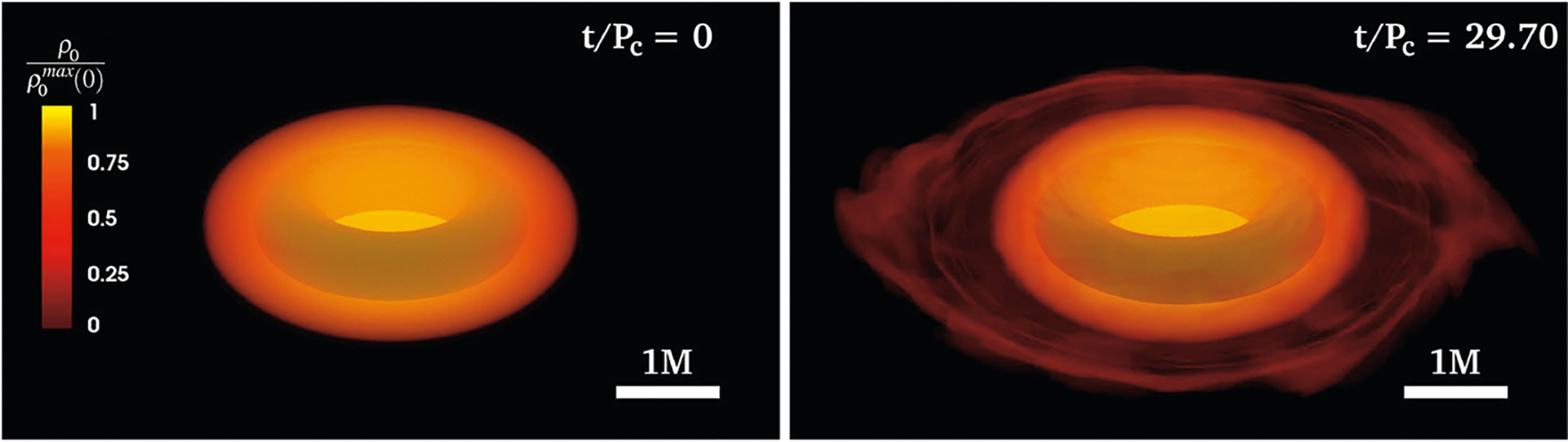
Initial and final profiles of a dynamically stable ergostar modeled with the ALF2cc EOS (see Equation 8). The rest-mass density *ρ*_0_ is normalized to its initial maximum value. The inner shaded torus indicates the position of the ergoregion. Here *P*_c_ is the initial rotation period measured at the point where the rest-mass density is maximum (from Tsokaros et al., 2019b).

Recently, Tsokaros et al. (2019b, 2020b) constructed the first dynamically stable ergostars using compressible, causal EOSs based on the ALF2 and SLy EOSs, but with their inner core replaced by the maximally stiff EOS in Equation (8). The solutions are highly differentially rotating HMNSs with a corresponding spherical compaction of C=0.3. In principle, such objects may form during NSNS mergers. Their stability was demonstrated by evolving them in full GR for over a hundred dynamical times (≳ 30 rotational periods) and observing their quasi-stationary behavior (see Figure 11). This stability was in contrast to earlier Γ = 3 polytropic models (Komatsu et al., 1989), which proved radially unstable to collapse (Tsokaros et al., 2019b).

**FIGURE 12 |:**
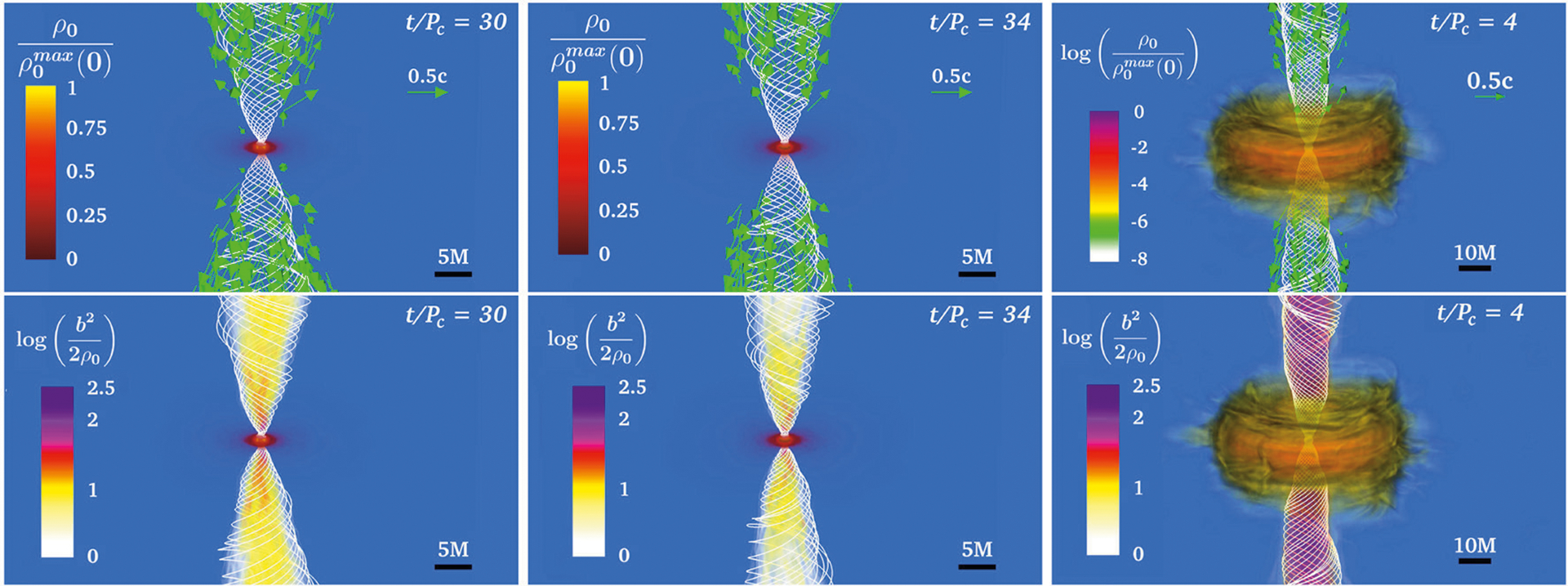
Final profiles of the rest-mass density *ρ*_0_ normalized to the initial maximum density **(top)**, and the force-free parameter inside the helical magnetic funnel **(bottom)** for a standard HMNS (left), an ergostar (middle row), and BH + disk (right). White lines depict the magnetic field lines, while the arrows display fluid velocities. *P*_c_ is the rotation period measure at the point where the rest-mass density is maximum. Here *M* = 5.9 km and *b*^2^ = *B*^2^/4 *π*. (from Ruiz et al., 2020c).

Using the above models, Ruiz et al. (2020c) performed the first fully GRMHD simulations of dynamically stable ergostars to assess the impact of ergoregions on launching magnetically–driven outflows. In addition, and for comparison purposes, the evolution of a standard magnetic HMNS without an ergoregion and a highly spinning BH surrounded by a magnetized accretion disk were also considered. The ergostar and the standard HMNS were initially endowed with a pulsar-like magnetic field generated by the vector potential in Equation (6), while the accretion disk was endowed with a poloidal magnetic field confined to the interior (see Equation 5). In all cases, after a few Alfvén times, the seed magnetic field is wound into a helical structure from which matter begins to flow outward (see Figure 12). In the HMNS cases (ergostar and standard star), the maximum Lorentz factor in the outflow is Γ_L_ ~ 2.5, while in the BH + disk case Γ_L_ ~ 1.3. Therefore, a mildly relativistic jet is launched whether or not an ergoregion is present. However, only in the BH + disk case does the force-free parameter reach *B*^2^/8*πρ*_0_ ≳ 100, whereby the outflow can be accelerated to Γ_*L*_ ≳ 100 as required by sGRB models (Zou and Piran, 2010). These simulations suggest that the BZ process only operates when a BH is present, though the Poynting luminosity in all cases is comparable. Further studies are required to confirm this tentative conclusion.
